# Noise-induced masking of hearing in a labyrinth fish: effects on sound detection in croaking gouramis

**DOI:** 10.7717/peerj.14230

**Published:** 2022-11-10

**Authors:** Isabelle Pia Maiditsch, Friedrich Ladich

**Affiliations:** 1 Department of Behavioral and Cognitive Biology, University of Vienna, Vienna, Austria; 2 Paul Scherrer Institut, Villigen, Aargau, Switzerland

**Keywords:** Vocalizing fish, White noise, Auditory evoked potentials (AEP), Hearing thresholds, Masking effect

## Abstract

An increasing level of anthropogenic underwater noise (shipping, drilling, sonar use, *etc*.) impairs acoustic orientation and communication in fish by hindering signal transmission or detection. Different noise regimes can reduce the ability to detect sounds of conspecifics due to an upward shift of the hearing threshold, a phenomenon termed masking. We therefore investigated the masking effect of white noise on the auditory thresholds in female croaking gouramis (*Trichopsis vittata*, Osphronemidae). We hypothesized that noise would influence the detection of conspecific vocalizations and thus acoustic communication. The auditory evoked potentials (AEP) thresholds were measured at six different frequencies between 0.1 and 4 kHz using the AEP recording technique. Sound pressure level audiograms were determined under quiet laboratory conditions (no noise) and continuous white noise of 110 dB RMS. Thresholds increased in the presence of white noise at all tested frequencies by 12–18 dB, in particular at 1.5 kHz. Moreover, hearing curves were compared to spectra of conspecific sounds to assess sound detection in the presence of noise in various contexts. We showed that masking hinders the detection of conspecific sounds, which have main energies between 1.0 and 1.5 kHz. We predict that this will particularly affect hearing of female’s low-intensity purring sounds during mating. Accordingly, noise will negatively affect acoustic communication and most likely reproductive success.

## Introduction

Various ecological factors negatively affect communication in animals by hindering signal transmission or detection. The ability to communicate effectively with conspecifics is an essential aspect in social interactions because many animals produce and detect sounds during agonistic behaviour, courtship, or foraging. A noisy environment could therefore hinder both signal transmission and signal detection, whereby a reduced signal reception might subsequently influence behavioural responses (Hawkins, 2011; Cole, 2013). Many studies on human and birds have provided important insights on the topic and showed that a single, simple measure can be used to estimate the effect of manmade environmental noises on the perception of communication signals (Brumm, 2004; Bielefeld, 2012); review by Dooling, Leek & Popper (2015). Birds, such as the wild fairy-wrens *Malurus cyaneus*, showed that background noise affected the response to alarm calls, probably due to acoustic masking rather than distraction or changes in vigilance (Zhou, Radford & Magrath, 2019). Traffic noise has the potential to produce sensory, behavioral, and physiological changes in birds and marine mammals. If the principle holds for species as diverse as birds or humans, it probably also applies for fishes (Dooling, Leek & Popper, 2015; Dooling et al., 2019; Erbe et al., 2016). This calls for assessing the actual impact of anthropogenic noise on sound communication in fishes (Hawkins, 2011; Popper & Hawkins, 2019).

There are many sources of underwater anthropogenic sounds in the oceans, lakes and rivers, and man-made noise is increasingly affecting signaling as well as social behaviour of aquatic animals. Ship or boat traffic, hydrodynamic power plants, seismic exploration and other artificial noise sources have different acoustical characteristics, and their rapidly increasing noise levels constitute a major challenge in the life of animals (Popper & Hawkins, 2019). For example, noise impairs courtship behaviour and breeding in cichlids and gobiids (de Jong et al., 2016; de Jong et al., 2018a; de Jong et al., 2018b; Butler & Maruska, 2020; Butler & Maruska, 2021). Moreover, sound communication is affected in various taxa such as toadfishes (oyster toadfish *Opsanus tau*: Luczkovich et al., 2016; splendid toadfish: *Sanopus splendidus*: Pyć et al., 2021; plainfin midshipman *Porichthys notatus*: Brown et al., 2021, Mackiewicz, Putland & Mensinger, 2021; Lusitanian toadfish *Halobatrachus didactylus*: Alves, Amorim & Fonseca, 2021), sweepers (captive bigeye *Pempheris adspersa*: Van Oosterom et al., 2016), gobies (painted goby *Pomatoschistus pictus* and two-spotted goby *Gobiusculus flavescens*: de Jong et al., 2016; de Jong et al., 2018b) or labyrinth fishes (croaking gourami *Trichopsis vittata*: Maiditsch & Ladich, 2022b). Numerous aquatic species rely on acoustic communication for social interaction, and additional studies, reviewed by *e.g.*, Ladich (2019), showed the negative impacts of anthropogenic noise on social behaviour and communication in fishes. Sound detection itself could also be affected by noise because increasing levels result in auditory masking, by which hearing thresholds rise in the presence of another sound (Hamilton, 1957; Tavolga, 1967; Tavolga, 1974; Chapman & Hawkins, 1973; Fay, 1974; Fay & Simmons, 1999; Erbe et al., 2016; Popper & Hawkins, 2019). Such threshold shifts have been reported in representatives of vocal and non-vocal fish families for many noise types including natural ambient, white noise or anthropogenic noise (Ladich, 2019).

Auditory thresholds have been measured in more than 100 fish species from various families covering different hearing sensitivities. These have mostly been conducted under quiet laboratory conditions and, in several of these species, in the presence of different noise types (Fay, 1988; Ladich & Fay, 2013). Masking can occur under relatively quiet conditions such as backwaters of rivers, lakes, ponds, or low-noise aquaria. The Atlantic cod *Gadus morhua*, for example, shows best hearing sensitivity under the quietest sea conditions, whereas masking occurs with any increase in the level of ambient sea noise (Chapman & Hawkins, 1973). The shifts are much more pronounced at higher noise levels, *e.g.*, in fast-flowing rivers and coastal surf. Masking by various ambient noise types has been investigated in several freshwater fishes (goldfish *Carassius auratus*: Enger, 1973; Fay, 1974; Gutscher, Wysocki & Ladich, 2011; common carp *Cyprinus carpio*, the European perch *Perca fluviatilis*: Amoser & Ladich, 2005; the topmouth minnow *Pseudorasbora parva*: Scholz & Ladich, 2006; the blacktail shiner *Cyprinella venusta*: Crovo et al., 2015; Holt & Johnston, 2015). Other studies showed an increase in hearing thresholds in the presence of boat noise and a reduced ability to detect conspecific vocalizations (*H. didactylus*: Alves, Amorim & Fonseca, 2021; Vasconcelos, Amorim & Ladich, 2007; different Mediterranean fish species: Codarin et al., 2009; meagre *Argyrosomus regius*: Vieira et al., 2021). White noise was used as a masker in cyprinids, centrarchids, sciaenids and cichlids (*C. auratus,* the Southern striped raphael catfish *Platydoras armatulus* and the pumpkinseed sunfish *Lepomis gibbosus:* Wysocki & Ladich, 2005a; Atlantic croaker *Micropogon undulatus* and black drum *Pogonias chromis*: Ramcharitar & Popper, 2004; orange chromide *Etroplus maculatus* and slender lionhead cichlid *Steatocranus tinanti*: Ladich & Schulz-Mirbach, 2013).

Importantly, the amount of masking depends not only on the noise level or noise type, but also on the hearing sensitivities. The term sensitivity generally refers to auditory perception of a sound by an individual, and it is likely that all fishes can detect sound (Lucke et al., 2016; Popper & Hawkins, 2019). Importantly, species with enhanced hearing abilities (hearing specialists) such as otophysans or some cichlids exhibit a higher responsiveness in detecting sound. Such species are more affected by noise than those lacking hearing enhancement (Ladich & Schulz-Mirbach, 2013; Ladich, 2019). The anabantoid fish *Trichopsis vittata* possesses an air-filled suprabranchial chamber for air-breathing laterally to the inner ears; this extends its hearing range (hearing specialists) up to several kHz and lowers the auditory thresholds over the entire frequency range (Schneider, 1964; Ladich & Yan, 1998). Both sexes of *T. vittata* vocalize loudly when defending territories, and females also vocalize prior to mating (Marshall, 1966; Ladich, 1998; Ladich, 2007). We chose the croaking gourami as a model species to better understand the detection of conspecific sounds in a noisy environment under standardized laboratory conditions.

The aim of the study is twofold: (1) we measured unmasked and masked hearing thresholds to determine the extent to which standardized white noise deteriorates the sound pressure sensitivity in a vocal hearing specialist, the anabantoid *T. vittata*; (2) we compared unmasked and masked hearing thresholds to the spectra of conspecific sounds. These comparisons will clarify the extent to which noise reduces the ability of *T. vittata* to detect conspecific sounds and correctly assess opponents and mates (Ladich, 1998; Ladich, 2007).

## Material and Methods

Animals were handled as described previously in Maiditsch & Ladich (2022a). Data were collected and analyzed with the method first described in Kenyon, Ladich & Yan (1998), Wysocki & Ladich (2002) and previously described in Ladich & Schulz-Mirbach (2013) and Maiditsch & Ladich (2014). Each paper is addressed in each method section where the method was first used.

### Animals

Ten adult female croaking gouramis were used for the experiments (body weight: 1.26–1.76 g, standard length: 38.8–47.3 mm), obtained from a local aquarium store in Vienna. Sexing of fish was based on the presence of the whitish ovary in females (Maiditsch & Ladich, 2022a). Females were chosen because of availability and because they do not differ from males in signalling during agonistic behaviour (Ladich, 2007; Ladich & Maiditsch, 2018; Maiditsch & Ladich, 2022a). All fishes were kept in community tanks (100 × 50 × 40 cm) at 25 ± 1 °C, with light maintained in a 12h:12 h light:dark cycle. Water was filtered by external filters. Holding tank bottoms were covered with sand and equipped with plants and halved flowerpots and tubes as hiding places. The fish were fed with frozen chironomid larvae or commercially prepared flake food (Tetramin) five times a week. No fish were euthanized or killed during or after the measurements. After the experiments all fish were returned to the community tanks (Maiditsch & Ladich, 2022a).

### Ethical considerations

All applicable national and institutional guidelines for the care and use of animals were followed (permit numbers BMWFW-66.006/0035-WF/V/3b/2017; Animal Ethic and Experimental Board, Faculty of Life Science 2017-010).

### Auditory evoked potential measurements

Auditory thresholds were measured using the auditory evoked potential (AEP) recording technique (first described by Kenyon, Ladich & Yan, 1998; Wysocki & Ladich, 2002). The test individuals were immobilized during the hearing experiments using Flaxedil (gallamine triethiodide; Sigma-Aldrich, Vienna, Austria). The average dosage used was 1.8 µg g^−1^(1−2.5 µg g^−1^) and enabled the fish to breathe during the experiment but with only slight opercula movement that prevented an excessive myogenic noise level, which could interfere with the recordings. Individuals that started to move prior to the end of the measurements were not immobilized a second time. This explains the different numbers of females measured at different frequencies (Table 1). For the AEP measurement, fish were secured in a round plastic tub (35 cm diameter; 15 cm height), the water temperature was maintained at 25 ±1 °C using a submersible heater, and the sides as well as the bottom were covered with a layer of bubble wrap and fine sand, because bubble wrap reduces reverberations (see Fig. 1 in Wysocki & Ladich, 2002).

The fish’s head was positioned just below the water surface and a respiration pipette was inserted into the animals’ mouth to allow respiration using a temperature-controlled gravity-fed circulation system. The plastic tub was positioned on an air table (TCM Micro-g 63-540), which rested on a vibration-isolated concrete plate. The entire setup was enclosed in a semi-soundproof room constructed as a Faraday cage (method was previously used and described in Maiditsch & Ladich, 2014). For AEP recordings, silver electrodes (0.32 mm diameter) were placed in the midline of the skull. The recording electrode was positioned over the region of the medulla and the reference electrode cranially between the nares; both were pressed firmly against the skin, which was covered with a small piece of Kim-wipes tissue paper to keep it moist; this ensured proper contact during experiments. Shielded electrode leads were attached to the differential input of a preamplifier (Grass P-55; Grass Instruments, West Warwick, RI, USA; gain 10,000x, high-pass at 30 Hz, low-pass at 1 kHz). A ground electrode was placed in the water. Stimuli presentation and AEP-waveform recording were specified using a modular rackmount system (TDT System 3; Tucker-Davis Technologies, Gainesville, FL, USA) running TDT BioSig RP Software (Maiditsch & Ladich, 2014; Ladich & Schulz-Mirbach, 2013).

**Table 1:** Mean (±SE) AEP hearing thresholds and number of female *T. vittata* measured at different frequencies and noise conditions and thresholds shifts between noise conditions. All thresholds in dB re 1 µPa.

Frequency (Hz)	No-Noise			White Noise			Differences		
	Mean	SE	N	Mean	SE	N	Mean	SE	N
0.1	96.4	1.04	10	108.1	0.14	10	11.7	1.11	10
0.5	92.4	1.01	10	104.3	0.83	10	11.9	1.29	10
1.0	90.2	1.95	10	103.1	0.96	9	12	2.07	9
1.5	85.5	0.82	10	104.1	1.20	9	18.4	1.03	9
2.0	92.1	1.24	10	105.3	1.08	10	13.2	1.21	10
4.0	108.7	2.16	10	120.6	0.84	7	15.6	1.86	7

### Sound stimuli

**Figure 1:**
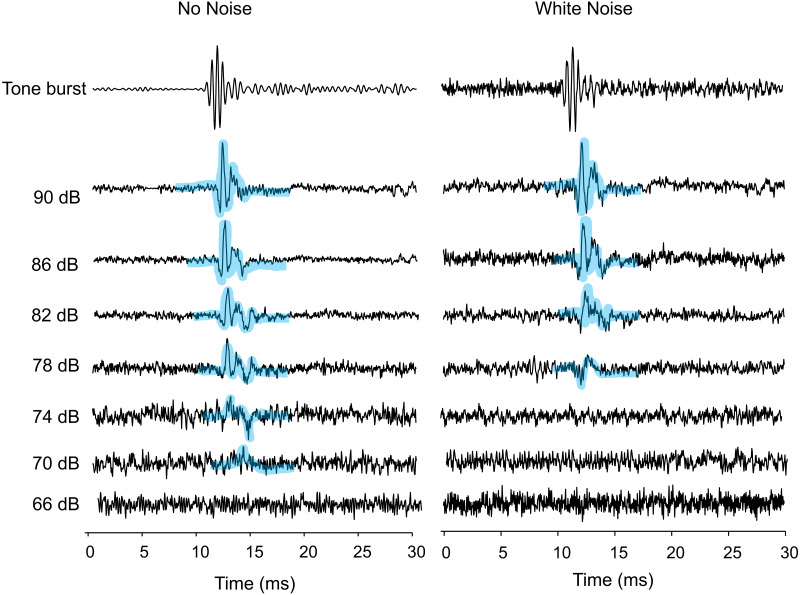
AEPs of *Trichopsis vittata* in response to a 2 kHz tone burst, in the presence of lab noise (No Noise left traces) and white noise (right traces). Tone burst levels were reduced in 4 dB steps until the AEP waveform was no longer visible. The hearing threshold during the No Noise condition was 70 dB. The threshold during the White Noise condition was at 78 dB. All SPLs are given in dB re 1 µPa. AEPs are highlighted by thick bluish transparent lines.

Sound stimuli were generated using TDT SigGen RP software and fed through a power amplifier (Alesis RA 300; Alesis Corporation, Los Angeles, CA, USA) to a dual-cone speaker (Tannoy System 600, frequency response 50 Hz to 15 kHz), which was placed 1 m above the tub. Sound stimuli were presented as tone bursts at a repetition rate of 21 per second. Hearing thresholds were determined at frequencies of 0.1, 0.5, 1, 1.5, 2 and 4 kHz, presented in random order. All bursts were gated using a Blackman window. The stimuli were presented at opposite polarities (180° phase shifted) for each test condition and the corresponding AEPs were averaged by the BioSig RP software in order to eliminate stimulus artefacts. The sound pressure level (SPL) of tone-burst stimuli was reduced in 4 dB steps until the AEP waveform was no longer apparent (Fig. 1). The lowest SPL for which a repeatable AEP trace could be obtained, which was determined by overlaying replicate traces, was considered the threshold. Particle motion thresholds were not measured because croaking gourami are hearing specialists and communicate with acoustic signals with main energies between 1 kHz and 2 kHz, a frequency range in which sound pressure is the relevant stimulus. Myrberg & Spires (1980) showed experimentally that fish are sound pressure sensitive above 300 Hz, while particle motion is the relevant stimulus at 100 and partly at 200 Hz.

To determine absolute SPLs of hearing thresholds at different frequencies, a hydrophone (Brüel & Kjaer 8101, −184 dB re 1 V/ µPa) was placed at the same position as the fish, after the AEP measurements (relative hearing thresholds at different frequencies with and without white noise). Using BioSig RP, the RMS voltage of the largest (*i.e.,* center) sinusoid of a particular tone-burst recording was determined. This RMS voltage was then used to calculate the absolute SPL re 1 µPa based on the sensitivity of the hydrophone and the amplification factor of the hydrophone amplifier and of the TDT system (Maiditsch & Ladich, 2014).

### Noise measurement and presentation

Audiograms were measured under normal laboratory conditions (91 dB re 1 µPa, RMS) and in the presence of continuous white noise played back at a sound level of 110 dB re 1 µPa (RMS) (method as described in Ladich & Schulz-Mirbach, 2013). We chose white noise at 110 dB because this level is within the low natural ambient noise range. The intent was to study general effects of noise on hearing and communication, not the effects of particular ambient noise types (Wysocki, Amoser & Ladich, 2007). Masking noise was created by Cool Edit 2000 (Syntrillium Software Corporation, Phoenix, AZ, USA) and sent *via* an external soundcard (Roland Rubix 22) to a 30-band equalizer (Alesis DEQ 230) to obtain a flat noise spectrum underwater and fed to the second channel of a signal mixer (SM5 of TDT System 3). The tone burst signals were fed to the first channel of the signal mixer. Both signals were then fed *via* the Alesis RA 300 amplifier to the dual-cone speaker (Tannoy System 600). The SPLs of the masking noise were measured at the position of the fish using the hydrophone, which was connected to a power supply (Brüel & Kjaer 2804) and a sound level meter (Brüel & Kjaer 2238 Mediator). We determined L-weighted (5 Hz to 20 kHz) equivalent continuous SPL (LLeq) averaged over 1 min measuring time. The Leq is a measure of the averaged energy in a varying sound level and commonly used to assess environmental noise. We also measured background noise levels in the experimental test tank (normal laboratory conditions). After SPL measurements, the background noise and the white masking noise were recorded *via* an external sound card (Cakewalk UA-25 EX) on a PC. Recording and analyzing were done using S_Tools-STX 3.7.8, an acoustics, speech, and signal processing application developed by the Acoustics Research Institute at the Austrian Academy of Sciences, Vienna. Sound spectra of 1 min recordings were calculated by an FFT analysis using a filter bandwidth of 1 Hz. Absolute spectral values were calculated from the relative spectral values (Wysocki & Ladich, 2005a; Ladich & Schulz-Mirbach, 2013).

### Sound spectra

The average SPL of the female croaking sound produced during dyadic contests and of purring sounds produced by females prior to mating were used to generate sound spectra. All sounds were recorded under laboratory conditions in previous studies (for details see Material and Method sections in Ladich, 2007 and in Ladich & Maiditsch, 2018).

### Statistical analysis

Differences between mean thresholds at different frequencies at no noise conditions were calculated using a one-way analysis of variance (ANOVA) and Bonferroni *post hoc* test.

Audiograms of the different experimental groups (no noise and white noise) were compared by two-factor analysis of variance (ANOVA) using a general linear model where one factor was noise-treatment and the other was frequency. The noise-treatment group factor alone should indicate overall differences between different treatments of animals, and in combination with the frequency factor if different tendencies exist at different frequencies of the audiograms. All calculations were done using SPSS 26 (IBM SPSS Statistics, Armonk, NY, USA).

## Results

**Figure 2:**
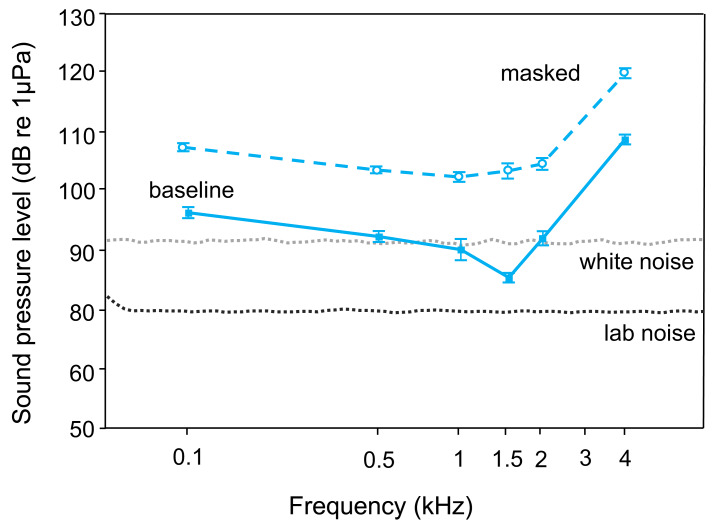
Mean (±S.E.) baseline (lower curve–solid line, filled square) and masked (upper curve–dashed line open circle) AEP thresholds of *T. vittata*. The lines below show the cepstrum-smoothed power spectra of the laboratory noise (lower dotted line–lab noise) and the white noise (upper dotted line–white noise 110 dB). All baseline thresholds shift upwards during playback of white noise.

The lab noise (No Noise) SPL-audiogram of *T. vittata* revealed best hearing ability at 1.5 kHz (average threshold: 86 dB re 1 µPa) and lowest sensitivity at 4 kHz (average threshold: 109 dB re 1 µPa) (Tables 1, 2, Fig. 2). During the white noise treatment, lowest AEP thresholds were found at 1 kHz (average threshold: 103 dB re 1 µPa). Mean hearing thresholds differed from 12–18 dB re 1 µPa between frequencies and treatments (Table 1).

Playback of white noise drastically lessened auditory sensitivity. Comparing audiograms by a two-factor ANOVA revealed significant overall differences (F1, 103 = 499.38, *p* < 0.001) and a significant interaction between noise condition and frequency (F5, 103 = 3.25, *p* < 0.01). Thus, changes in thresholds showed different trends at different frequencies.

### Sound detection by conspecifics

**Figure 3:**
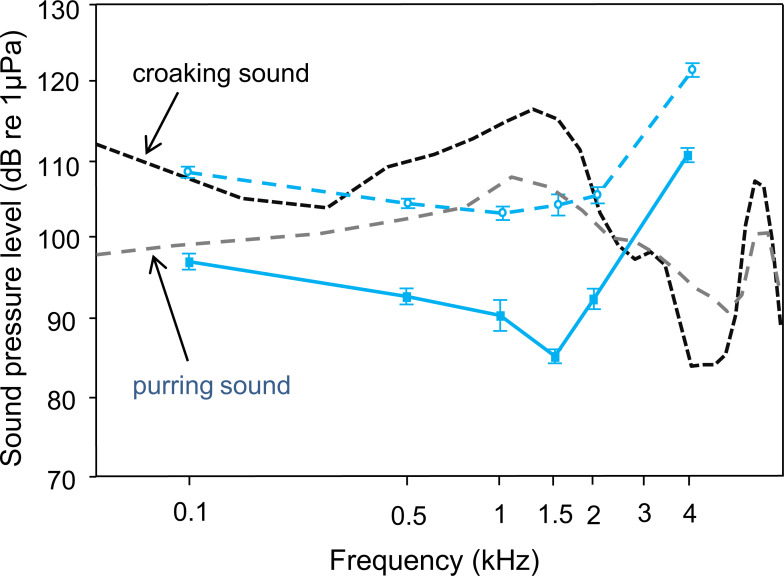
Mean (±S.E.) hearing threshold of *T. vittata*; during ambient laboratory noise (lower curve–red solid line, filled square) and continuous white noise (upper curve–red dashed line, open circle), compared to sound power spectra of a typical croaking sound with its main energy at 1.5 kHz (black dashed line and a pre-spawning purring sound with its main energy at 1.25 kHz (dashed green line). Detectability of both sound types decreases when hearing thresholds shift upwards.

Comparisons between croaking and pre-spawning purring sound spectra and the ambient noise audiogram indicated that *T. vittata* could detect sounds under quiet laboratory conditions (Fig. 3). The sound energy was more than 30 dB above the baseline hearing thresholds at the most sensitive frequency of 1.5 kHz, where the main energies of sounds are concentrated. Under continuous white noise conditions, the sound energy of a croaking sound is maximally 15 dB above the masked AEP thresholds. The pre-spawning purring sound, which is lower in SPL, is still detectable under no noise conditions, but under white noise conditions merely at a communication distance of a few centimetres.

**Table 2:** Differences between mean auditory thresholds at different frequencies in dB at no noise condition.

Frequency (Hz)	0.1 kHz	0.5 kHz	1 kHz	1.5 kHz	2 kHz	4 kHz
0.1 kHz	–	4.0	**6.2**	**10.9**	4.3	**12.3**
0.5 kHz	4.0	–	2.2	6.9	0.3	**16.3**
1 kHz	**6.2**	2.2	–	4.7^*^)	1.9	**18.5**
1.5 kHz	**10.9**	**6.9**	4.7^*^)	**–**	**6.6**	**23.2**
2 kHz	4.3	0.3	1.9	6.6	–	**16.6**
4 kHz	**12.3**	**16.3**	**18.5**	**23.2**	**16.6**	**–**

**Notes.**

Bold numbers: significant differences between thresholds. *) one-tailed.

## Discussion

### Effects of noise on AEP thresholds

**Figure 4:**
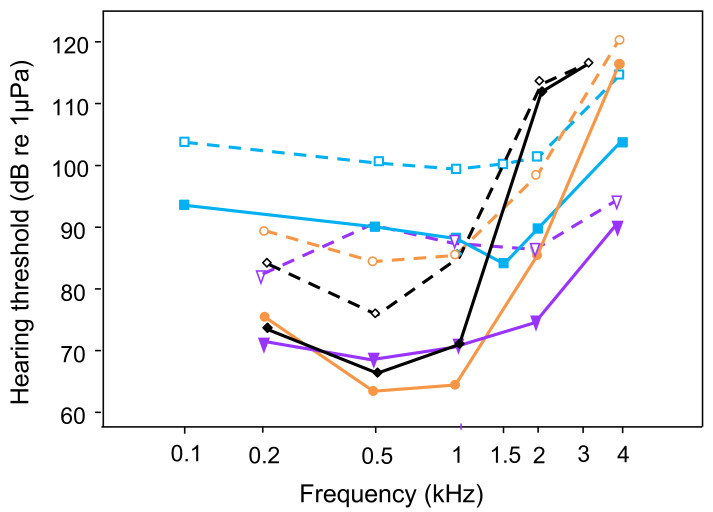
Comparison of AEP-hearing thresholds in fish species possessing accessory hearing structures (hearing specialists); under laboratory noise (solid lines and filled symbols) and continuous white noise (dashed lines and open symbols) at 110 dB: *Trichopsis vittata* (blue lines -■-, current study); *Carassius auratus* (orange lines -•-, Wysocki & Ladich, 2005a), *Platydoras armatulus* (purple lines -▾-, Wysocki & Ladich, 2005a); *Etroplus maculatus* (black lines -◊-, Ladich & Schulz-Mirbach, 2013). White noise results in threshold shifts at the most sensitive frequencies in all hearing specialists.

The present study shows that continuous white noise of 110 dB (RMS) significantly increased the auditory thresholds, and that this masking effect was maximal within the most sensitive hearing range of *T. vittata*, between 1 and 2 kHz. Moreover, masking of thresholds was more pronounced at the upper ends of the audiogram’s frequency range. These findings agree other studies using the same AEP threshold measuring technique and identical noise conditions (white noise of 110 dB RMS), but conducted on fish from different orders possessing hearing specializations (hearing specialists, Popper, Hawkins & Sisneros, 2021). A white noise level of 110 dB resulted in a threshold shift throughout the frequency range in particular at the most sensitive frequencies (Fig. 4): 20 dB at 0.5 and 1 kHz in *C. auratus*, 22 dB at 0.5 kHz in *P. armatulus* (Wysocki & Ladich, 2005a) and 11 dB at 1 kHz in the cichlid *E. maculatus* (Ladich & Schulz-Mirbach, 2013). This indicates that noise similarly limits sound detection and thus acoustic orientation in all hearing specialists. An increase in white noise level to 130 dB elevated overall hearing thresholds further in otophysines and in the cichlid *E. maculatus* (Wysocki & Ladich, 2005a; Ladich & Schulz-Mirbach, 2013). Similar results were reported by Ramcharitar & Popper (2004) in sciaenids, where white noise at 124 dB altered auditory sensitivity in the black drum and the Atlantic croaker. Especially the black drum was no longer able to detect signals at the highest frequency of its detection range. Increasing the masking level to 136 dB triggered even greater shifts in auditory thresholds in the black drum, particularly in the frequency range 300–600 Hz.

Fishes live in environments with highly diverse acoustic backgrounds in terms of both noise levels and energy distribution (Chapman & Hawkins, 1973; Wysocki, Amoser & Ladich, 2007). Several earlier masking studies using different paradigms showed similarly that noise masks hearing in different fish species. Studies on cods, goldfish, pin fish *Lagodon rhomboides* and the African mouth-breeder *Tilapia macrocephaIa* revealed that hearing thresholds depend on background noise levels at particular frequencies (Buerkle, 1968; Buerkle, 1969; Fay, 1974; Tavolga, 1974). Buerkle (1968) stated that they varied directly with background noise and that signal-to-noise levels at threshold remained approximately the same at all background noise levels. Similar threshold shifts were subsequently revealed using different noise types such as white noise, ambient noise in the field (or played back in the lab) as well as by noise in artificial environments such as aquaria or aquacultural facilities (Chapman & Hawkins, 1973; Amoser & Ladich, 2005; Gutscher, Wysocki & Ladich, 2011).

How do these observations and current results relate to hearing in croaking gouramis in their natural habitats? This species inhabits shallow, still, densely vegetated waters in South-East Asia. Labyrinth fishes overcome the lack of oxygen in such still tropical waters by airbreathing using the suprabranchial chamber dorsally of the gills (Bader, 1937). Still waters allow gouramis to build bubble nests on the water surface for their brood. A comparison of 12 aquatic habitats by Wysocki, Amoser & Ladich (2007) demonstrated that the natural habitats of *T. vittata* are rather quiet compared to running waters. We therefore assume that this species lives under noise conditions similar to the no noise condition in the lab. Anthropogenic noise, however, can mask hearing in shallow waters as well. Holt and Johnston (2005) showed that traffic noise from streets may masks acoustic signaling in fish such as shiners (family Cyprinidae) inhabiting shallow freshwater streams.

### Sound detection

The present study and that of Ladich & Yan (1998) underline that croaking sounds are clearly detectable by both sexes of *T. vittata* under laboratory noise conditions. The spectral sound energy was at least 20 dB above the baseline hearing thresholds at the most sensitive frequency of 1.5 kHz. Hearing provides fishes with important information in a broad range of environments, making sound a key cue for perhaps most aquatic animals (Holt & Johnston, 2014; Popper & Hawkins, 2019). Noise may therefore reduce the acoustic active space and affect the social and acoustic behaviour in fish, with potential consequences on courtship and breeding behaviour. This would particularly impact territorial animals that are unlikely to leave their site, even in disadvantageous conditions (Butler & Maruska, 2020). Using tonal noise to simulate a noisy environment, male *Astatotilapia burtoni* showed fewer territorial fights and spawning: noise-exposed *A. burtoni* females, which had significantly higher auditory thresholds (in their most sensitive frequency range: 100 and 200 Hz), probably failed to detect male vocalizations during courtship. In croaking gouramis, both sexes emit croaking sounds during dyadic contests, which differ only in SPL because male sounds are 4-5 dB louder than those of females. In contrast, only females produce purring sounds prior to spawning, which are only half as long and half as loud as their croaking sounds (6 dB lower in SPL) (Ladich, 2007). We assume that the hearing sensitivities are similar in both sexes, so that male and female hearing could be similarly masked. Loud croaking sounds may be similarly detectable by both sexes under the noise conditions played back in this study.I Importantly, however, masking of hearing thresholds could lead to erroneous assessments of an opponent’s fighting abilities (body size) and subsequently affect reproductive success (Fig. 3). Note that acoustic communication takes place at a distance of 1–5 cm in croaking gouramis. Thus, levels of croaking sounds measured in this and prior studies at a distance of 10 cm are lower than those detected by fish during fighting and mating at a shorter distance. Nevertheless, we assume that low-intensity purring sounds will not be detected by merely be detected by males during mating in noisy environments. This could affect mate assessment and affect both fitness and reproductive success.

Similar findings in the male Lusitanian toadfish demonstrate that the detectability of their boatwhistle calls decreased considerably in the presence of noise: sound frequencies were detectable only up to 300 Hz, indicating that noise decreases the ability of females to find nest sites of calling male nest sites and assess mates (Vasconcelos, Amorim & Ladich, 2007). In the damselfish *C. chromis* as well as the brown meagre, conservative calculations regarding the distance at which conspecific sounds are detectable (brown meagre) yielded brief decreases from 500 m under ambient noise conditions to about 1 m in the presence of noise (Codarin et al., 2009; Ladich, 2019). Moreover, sound detection also depends on the habitat, time of day, and season. The topmouth minnow *P. parva*, a hearing specialist, produces loud sounds while feeding, which can be detected under ambient noise conditions up to 0.4 m. Scholz & Ladich (2006) assume that in the minnow’s natural habitat, feeding sounds would be more difficult to detect in the presence of recreational activities (*e.g.*, boating, surfing, swimming). Together with the current study, these data indicate that noise does influence the hearing ability in fish and may therefore negatively impact communication distances, reproductive success, and aquatic ecosystems in general.

### An upcoming problem: traffic noise and auditory ability

In general, an upcoming problem in natural environments is the masking effect due to the expansion of traffic noise, ship noise, and aquatic industrial activities. All have increased in recent years and have led to concern about the effects of man-made sounds on aquatic life (Hawkins, Pembroke & Popper, 2015). Birds experience threshold shifts when exposed to noise, but even if they remain close to high levels of traffic or urban noise sources, this is unlikely to cause permanent hearing loss or auditory damage. Some species even showed strategies to improve their communication space, European black-birds (*Turdus merula*) and great tits (*Parus major*) apparently benefit from closing the distance between them, simply by moving upward to a higher perch (Dooling et al., 2019). In several marine mammals and fish, communication signals often occur in the same frequency range as vessel noise and audiograms. Their threshold ranges overlap with those of vessel noise, making these animals susceptible to auditory masking. Ship noise clearly impacts fish. Codarin et al. (2009) showed in three Mediterranean species (damselfish *Chromis chromis*, brown meagre *Sciaena umbra* and red-mouthed goby *Gobius cruentatus*) an increase of hearing thresholds by approximately 20 dB in the presence of boat noise, but no such effect under either quiet laboratory or ambient noise conditions. Similar observations were made in the Lusitanian toadfish: the main energies of ferry-boat noise were within the most sensitive hearing range and increased its auditory threshold by up to 36 dB at most frequencies tested. This indicates that acoustic communication is affected by masking of their hearing abilities (Vasconcelos, Amorim & Ladich, 2007). In the meagre, Vieira et al. (2021) evaluated how noise from a ferry-boat and a small boat with an outboard engine impacted hearing ability. Boat noise produced a masking effect and increased the detection threshold by 20 dB. In this case, the reduced ability of juvenile meagre to discriminate conspecific calls would be equivalent to an approximate 90% reduction in communication space. The conclusion is that significant consequences for individuals but also populations are possible as a result of altered acoustic behaviour due to anthropogenic noise. Even levels far lower than those that induce mortality could cause physiological changes, changes in behavior, and mask biologically important sound (Popper & Hawkins, 2019).

## Conclusion

This is the first study to investigate the effect of noise on the AEP thresholds in the croaking gourami, a highly vocal labyrinth fish species. We know from a former study (Maiditsch & Ladich, 2022b) that noise does not affect the *amount* of acoustic and visual signaling during agonistic behaviour. But *T. vittata* is unable to adapt its sound characteristics, and our conclusion is that noise can therefore negatively affects sound detection by masking the thresholds of female *T. vittata*. This would impact acoustic communication during agonistic and mating behaviour and affect the assessment of opponents and mates during territory defense and reproduction. A future study on the effect of noise on males would help clarify the extent to which noise impacts their hearing ability and potentially influences reproduction success.

##  Supplemental Information

10.7717/peerj.14230/supp-1 Supplemental Information 1 Raw data of hearing thresholds per individual measured at different frequencies and noise conditions All SPLs are given in dB re 1 µPa.

10.7717/peerj.14230/supp-2 Supplemental Information 2 ARRIVE 2.0 Checklist

## Additional Information and Declarations

### Data Availability

The following information was supplied regarding data availability: The raw measurements are available in the Supplemental Files.
